# Effects of dance training on oxytocin secretion and neural activity in older adults with subjective cognitive decline

**DOI:** 10.1093/geroni/igaf129

**Published:** 2025-11-14

**Authors:** Masatoshi Yamashita, Aya Toyoshima, Shoko Iwasaki, Reina Takamatsu, Hiroyuki Muto, Nobuhito Abe, Jin Narumoto, Kaoru Sekiyama

**Affiliations:** Research Center for Child Mental Development, University of Fukui, Eiheiji, Fukui Prefecture, Japan; United Graduate School of Child Development, Osaka University, Kanazawa University, Hamamatsu University of Medicine, Chiba University, and the University of Fukui, Suita, Osaka Prefecture, Japan; Faculty of Human Sciences, Shimane University, Matsue, Shimane Prefecture, Japan; Graduate School of Letters, Kyoto University, Kyoto, Kyoto Prefecture, Japan; Faculty of Policy Studies, Aichi Gakuin University, Nisshin, Aichi Prefecture, Japan; Graduate School of Sustainable System Sciences, Osaka Metropolitan University, Sakai, Osaka Prefecture, Japan; Institute for the Future of Human Society, Kyoto University, Kyoto, Kyoto Prefecture, Japan; Department of Psychiatry, Graduate School of Medical Science, Kyoto Prefectural University of Medicine, Kyoto, Kyoto Prefecture, Japan; Wildlife Research Center, Kyoto University, Kyoto, Kyoto Prefecture, Japan; Faculty of Social Informatics, ZEN University, Zushi, Kanagawa Prefecture, Japan

**Keywords:** Resting-state fMRI, Leisure activity, Amplitude of low-frequency fluctuations

## Abstract

**Background and Objectives:**

Subjective cognitive decline (SCD) is a preclinical stage of mild cognitive impairment (MCI). Although dance training has been shown to be beneficial for mental health, cognitive function, and neural activity in older adults with MCI, its effect on SCD remains unclear. This study aimed to examine the effects of dance training on the aforementioned factors and on oxytocin secretion in older adults with SCD.

**Research Design and Methods:**

Participants (aged 65–84 years) were assigned to either the intervention group (*n* = 22) with a 12-week dance training program or the control group without any alternative training (*n* = 22). Apathy, depression, Montreal Cognitive Assessment scores, urinary oxytocin levels, and resting-state functional magnetic resonance imaging indices, including amplitude of low-frequency fluctuations (ALFF) and functional connectivity (FC), were evaluated pre- and post-intervention.

**Results:**

Compared to the control group, the intervention group exhibited significantly higher urinary oxytocin levels and significantly higher ALFF in the left medial orbitofrontal cortex post-intervention. Moreover, the intervention group showed more enhanced FC between the left medial orbitofrontal cortex and the left precuneus post-intervention than the control group. However, mental health or cognitive performance was not significantly different between the groups.

**Discussion and Implications:**

Our results are particularly important in light of previous findings that older adults with SCD show a reduced FC between the medial orbitofrontal cortex and the precuneus, and that oxytocin levels are positively associated with the prefrontal-amygdala oxytocinergic circuit in socioemotional processing. Thus, dance training may contribute to socioemotional resilience-related neural and molecular adaptations in SCD.

Innovation and Translational Significance: As the global burden of dementia increases with population aging, there is a need to identify effective non-pharmacological interventions to mitigate the reductions in psychological well-being and brain function in subjective cognitive decline, a preclinical symptom of mild cognitive impairment and dementia. This study is the first to show that dance engagement increases oxytocin levels, spontaneous neural activity in the medial orbitofrontal cortex, and resting-state functional connectivity between the medial orbitofrontal cortex and precuneus in older adults with subjective cognitive decline, highlighting the potential of dance to promote socioemotional well-being in the early stages of cognitive decline.

Dementia, including Alzheimer’s disease, is a growing concern in Asia, particularly in aging countries (i.e., Japan). The onset of clinical symptoms of dementia can be preceded by both subjective and objective cognitive decline many years earlier (“2015 Alzheimer’s disease facts and figures,” 2015; Lin et al., 2019). Subjective cognitive decline (SCD) is characterized by a self-experienced decline in cognitive function (i.e., memory) compared to a previously normal cognitive status, while performance on standardized cognitive tests used to classify mild cognitive impairment (MCI) remains within the normal range (Jessen et al., 2020). Importantly, SCD is considered a preclinical symptom of MCI and dementia (Pike et al., 2022). Older adults with SCD show lower psychological well-being, higher levels of depression, and more impaired memory and metacognition than those without SCD (Fastame, 2022; ­Rotenberg & Dawson, 2022). These behavioral features may be accompanied by alterations in neural systems supporting self-referential and cognitive processes, particularly the default mode network with key hubs in the precuneus and medial prefrontal cortex (Gangopadhyay et al., 2021; Gilboa et al., 2004; Porcelli et al., 2019; Ye et al., 2019). In healthy older adults, functional connectivity (FC) between the anterior (i.e., medial prefrontal cortex) and posterior (i.e., posterior cingulate and precuneus) regions within the default mode network declines with age and is associated with cognitive decline (Andrews-Hanna et al., 2007; Ng et al., 2016). Extending this age-related pattern to SCD, older adults with SCD show a lower amplitude of low-frequency fluctuation (ALFF) in the precuneus and reduced FC between the precuneus and medial prefrontal cortex than those without SCD during resting-state functional magnetic resonance imaging (fMRI) (Li et al., 2024). Accordingly, changes in resting-state neural activity within the default mode network may be relevant not only to cognition but also to emotional regulation. Given these issues in a progressively aging society, it is important to identify effective non-pharmacological interventions to mitigate neuropsychological (i.e., cognition), neuropsychiatric (i.e., mental health), and neurobiological anomalies in the SCD stage.

Dance is associated with a lower risk of dementia (Verghese et al., 2003) and is thus a potentially effective non-pharmacological intervention for mitigating neuropsychological, neuropsychiatric, and neurobiological issues. Dance comprises complex physical and mental operations, such as rhythmic motor coordination, extremity movement in conjunction with acoustic stimulation, execution of imaged extremity movement, postural control, and the learning and recall of movement sequences (Hashimoto et al., 2015; Kattenstroth et al., 2013). Moreover, dancing promotes social interactions (i.e., interpersonal cooperation, feelings of togetherness, and group cohesion) and emotional cohesion through affective transmission (Hagen & Bryant, 2003; Nadasen, 2008). Given these multimodal characteristics, dance is a potentially beneficial intervention for improving mental health and cognitive function. Several studies have demonstrated that older adults with MCI who received dance training exhibited improved mental health (i.e., lower apathy and depression scores) and enhanced cognitive performance (i.e., higher Montreal Cognitive Assessment [MoCA] scores) (Dominguez et al., 2018; Wang et al., 2021; Yuan et al., 2022). Moreover, Sanprakhon et al. (2025) reported that in older adults with mild behavioral impairment (MBI), a 7-week multicomponent program combining traditional Thai folk dance with cognitive stimulation therapy was associated with reductions in the behavioral impairment total score (i.e., apathy) and cognitive failures questionnaire total score (i.e., everyday cognitive lapses), as well as improvements in MoCA performance. However, because these studies primarily focused on individuals with MCI or MBI, it remains unclear whether dance improves mental health or cognition in older adults with SCD, and the neurobiological mechanisms underlying such effects have not been directly examined.

In general, dance facilitates social interactions and emotional cohesion, and thus, it may also influence the neurochemical mechanisms that regulate social bonding. One such mechanism involves oxytocin, a neuropeptide known for its influence on prosocial behavior and emotional well-being (Harari-Dahan & Bernstein, 2014; Harvey, 2020; Heinrichs et al., 2003). For example, Josef et al. (2019) reported that during dance, participants who received intranasal oxytocin had better movement synchrony in dance pairs than did those who did not. Furthermore, the effects of oxytocin on social synchrony may be associated with activity in the prefrontal cortex and amygdala (Harvey, 2020). These findings indicate that dance training may enhance social synchrony through its effects on oxytocin levels, which may be mediated by brain regions associated with prosocial behavior and emotional processing. However, it remains unclear whether dance training increases endogenous oxytocin levels, and this question has not been examined in older adults with SCD.

Dance interventions have been found to affect brain activity. A previous study reported that healthy older adults showed increased activation in the supplementary motor cortex and cerebellum during the face-name associative memory task after a 6-week dance intervention (Ji et al., 2018). Although this finding suggests enhanced activation of motor- and working memory-related brain regions with dance training, the absence of a control group in the single-group pre-post design precludes ruling out practice effects. Additionally, Qi et al. (2019) reported that during resting-state fMRI, older adults with MCI show higher ALFF in the front-temporal regions, anterior cingulate cortex, and parahippocampal cortex after a 12-week dance intervention. With their liberal statistical threshold, the increase in spontaneous neural activity was observed not only in executive function-related areas but also in prosocial behavior-related areas (i.e., prefrontal cortex and anterior cingulate cortex) (Apps et al., 2016; Bos et al., 2012; Friedman & Robbins, 2022), suggesting a potential modulation of cognitive flexibility and social engagement in MCI in the context of dance training. However, the mechanisms through which dance interventions affect ALFF in older adults with SCD are yet to be clarified.

Collectively, evidence from older adults with MCI indicates that dance training can improve well-being, cognitive function, and brain activity. However, most studies have focused on MCI, whereas the neurobiological mechanisms of older adults with SCD have received scant attention. Given that SCD often precedes MCI and dementia and is characterized by socioemotional vulnerabilities and default mode network alterations, it is important to clarify whether dance engagement at this stage is effective in psychological well-being and brain function.

This study aimed to investigate whether a 12-week dance program could improve mental health, cognitive performance, urinary oxytocin levels, and resting-state neural activity in older adults with SCD.

## Research design and methods

### Study design

This study adopted a prospective, parallel-group, randomized controlled trial design. The trial was approved by the Kyoto University Graduate School and Faculty of Medicine Ethics Committee (approval number C1545) and conducted in Kyoto, Japan. All study participants provided their written informed consent, and the procedures complied with the principles of the Declaration of Helsinki. The trial was registered in the UMIN Clinical Trials Registry (UMIN000047106). This study reports prespecified outcomes (behavior measures, urinary oxytocin, and resting-state neural activity) from a single randomized controlled trial under one protocol and cohort. Additional modalities collected under the same protocol (i.e., structural MRI, task-based fMRI, and monoamine assays) will be analyzed and reported separately to maintain focus and interpretability. No prior publications on this cohort exist.

### Participants

**Figure 1.**
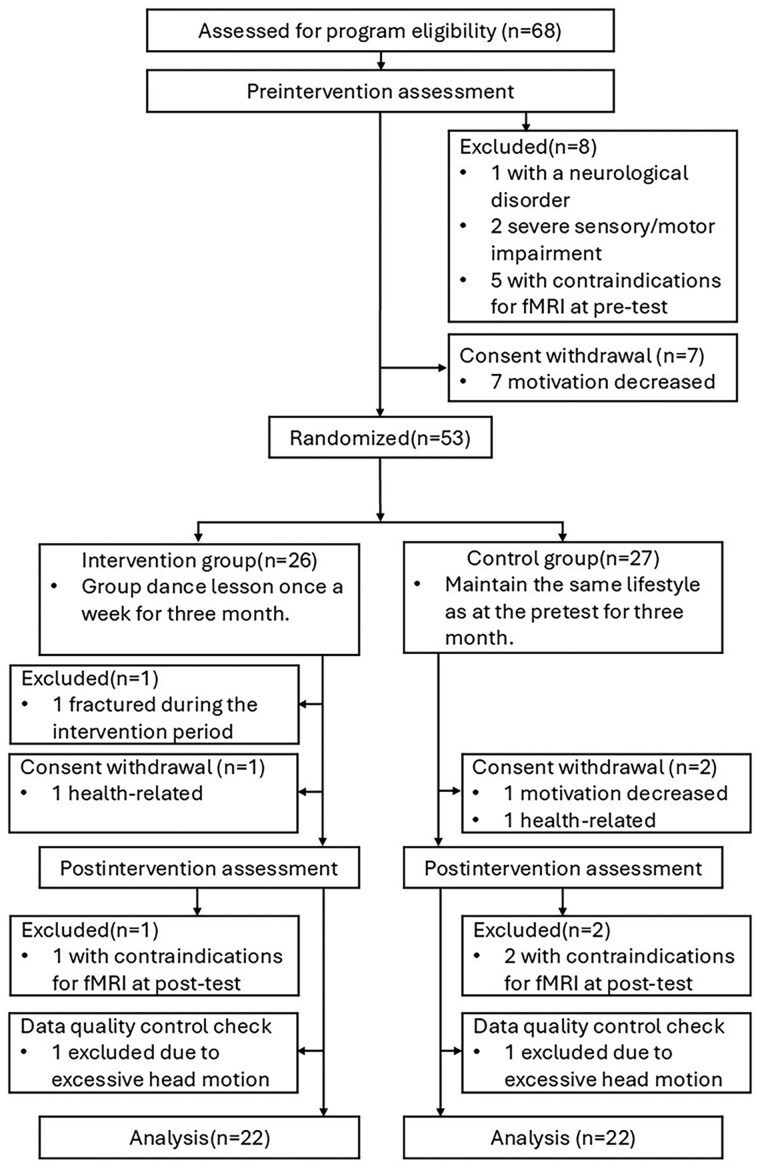
Participant selection flowchart.

This study included 68 independent community-dwelling older adults with SCD aged 65–84 years. In accordance with a previous study (Jessen et al., 2020), the inclusion criteria were as follows: (1) reporting worse forgetfulness compared to a year ago, (2) without a diagnosis of dementia, (3) independent living (i.e., Lawton Instrumental Activities of Daily Living Scale score ≥ 6; Lawton & Brody, 1969), and (4) able to travel to testing sites and attend dance classes. The exclusion criteria were as follows: (1) claustrophobia or panic disorder, (2) suspected dementia (i.e., Mini-Mental State Examination [MMSE] score ≤ 23 and MoCA score ≤ 18), (3) heart disease, (4) neurological disorder, and (5) medical history of mental disease. During recruitment, all participants were interviewed to confirm the absence of a formal diagnosis of dementia and to assess their functional and cognitive status. Specifically, instrumental activities of daily living were evaluated using the Lawton Instrumental Activities of Daily Living Scale, and cognitive functioning was preliminarily assessed through pretest scores on the MoCA or MMSE. For details of the participant flow, see the *Results* and Figure 1.

Participants were recruited through two local clinics by placing flyers in waiting areas. However, due to limited access during the COVID-19 pandemic, additional flyers were distributed directly to households in the surrounding community. Clinic staff were not involved beyond allowing the placement of flyers. Only two participants were recruited via clinic referrals; the rest responded to flyers. No monetary compensation was provided; participants received free access to dance lessons. Recruitment was conducted for approximately one month, and an intervention that included a pretest, dance practice, and posttest was conducted for approximately 5–6 months in a single period. The time between the pretest and the start of the intervention varied depending on participant scheduling, ranging from approximately 1 to 2 months. Posttest assessments were conducted within approximately 2 weeks after the final dance session, with this interval being consistent across participants. The recruitment process was repeated until sufficient samples were collected. This study consisted of five periods: (1) May 2022–September 2022; (2) July 2022–December 2022; (3) October 2022–April 2023; (4) February 2023–July 2023; and (5) October 2023–February 2024. The per-phase randomized and analyzed sample sizes (intervention/control) are summarized in Supplementary Table 1 (see online supplementary material). Seven participants who withdrew before randomization during the pre-intervention assessments were not attributed to either arm. After the pretest, participants were assigned to either the intervention or control group using adaptive randomization to balance demographics (i.e., age) and the Japanese version of the MoCA scores. Investigators were blinded to group assignments. The intervention group attended dance lessons once a week for 12 weeks. The control group did not receive dance-related lessons during the intervention phase and was offered dance lessons after the study period. Both groups were instructed to maintain the same lifestyle as in the pretest and not to start any new activities or adopt new lifestyle habits during the intervention phase (except the dance lesson for the intervention group); usual activities were not restricted.

### Intervention program

Dance practice was conducted once a week for 60 min for 12 weeks in a rental studio. The dance lessons were conducted by a commissioned instructor who had experience coaching dance practice with older adults in nursing homes. The participants engaged in learning choreographic pieces, one of which was approximately 2 min long and utilized familiar music from their generation (pop and Japanese traditional lyrics). They then repeatedly danced these learned routines. In each dance class, there were at least four participants.

Each session began with a brief self-introduction, followed by warm-up exercises, such as stretching, rhythmic exercises, humming songs, and walking to music. Thereafter, the participants received a lecture on choreographed movements to the lyrics of the music chosen by the instructor and danced in sync with the songs. A choreographed dance consisted of simple steps and arm movements. The tempo of the selected songs was approximately 80–120 beats per minute (BPM). The participants learned the choreography sections by sections, first to the counts repeatedly, and later to the music. Eventually, they continuously danced each of the entire song from the beginning to the end. Emphasis was placed on rhythmic accuracy. The participants learned the choreography of several songs over 12 sessions. All intervention groups began by practicing the same song, which had a tempo of 95 BPM, and spent approximately six sessions practicing choreography for the first song. In the remaining six sessions, they also practiced dancing to several other songs with tempos ranging from 80 to 120 BPM.

### Lifestyle questionnaire

The lifestyle questionnaire (Guo et al., 2020; Yamashita et al., 2022) is a 5-item questionnaire used to measure the current weekly frequencies (0–7 days/week) of exercise (e.g., walking, muscle training, calisthenics), cognitive activity (e.g., reading, crafts, painting), paid work, and volunteering. In addition, the outing frequency questionnaire was conducted (see Supplementary Methods [see online supplementary material]).

### Mental health and cognitive evaluations

Mental health was assessed using the Japanese version of the Center for Epidemiologic Studies-Depression (CES-D) Scale and the Apathy Evaluation Scale (AES-I-J) questionnaires. The CES-D (Shima et al., 1985) is widely used in surveys targeting communities and populations and is one of the most widely adopted depression scales worldwide. The CES-D consists of 20 items that assess depressive symptoms using a total score. Assessing the severity of depression based on the number of days with symptoms in the past week, the score is rated from “none,” “1–2 days,” “3–4 days,” to “5 days or more,” with scores ranging from 0 to 60 points. The AES-I-J (Kasai et al., 2014; Marin et al., 1991) is an apathy assessment tool for patients with dementia and is widely used internationally. The Japanese version consists of 18 items and is assessed using a total score. Participants were asked about their feelings and behaviors over the past 4 weeks, using a four-point scale: “none,” “slightly present,” “somewhat present,” and “very present.”

Cognitive function was assessed using the Japanese version of the MoCA. The MoCA (Fujiwara et al., 2010; Nasreddine et al., 2005) is a one-page, 30-point test that demonstrates high internal consistency and validity. Internal subtests of the MoCA were used to evaluate the following six cognitive domains: memory (5 points): delayed recall; executive function (4 points): letter fluency, trail making, verbal abstraction; visuospatial (4 points): cube copy, clock drawing; languages (5 points): naming, sentence repetition; attention (6 points): digit span forward and backward, letter A tapping, serial-7 subtraction; and orientation (6 points): orientation of date and place. One point was added to the total score of participants with less than 12 years of education to adjust for educational background.

### Chemiluminescent enzyme immunoassay for oxytocin

Oxytocin levels were measured from urine samples using a chemiluminescent enzyme immunoassay. On the day before testing, the participants were instructed to refrain from intense physical activity and consuming certain foods and beverages that can be difficult to digest (i.e., alcohol, coffee, high-fat fish, red beef, and blue cheese) for 12 hr. Between 12:30 and 14:30 of the test day, fresh urine samples were collected after a 10-min rest, stored at –80°C, and sent to a commercial laboratory (ASKA Pharmaceutical Medical Inc., Co., Ltd, Fujisawa City, Kanagawa, Japan). In brief, urine was deproteinized with acetonitrile containing 0.1% trifluoroacetic acid. Thereafter, the eluate was evaporated and extracted using a MonoSpin C18 reversed-phase column (s-type; GL Science). The samples were added to goat anti-rabbit immunoglobulin G antibody-precoated wells with alkaline phosphatase-conjugated oxytocin and rabbit antibodies specific to oxytocin and incubated at 4°C for 1 day. After washing the wells, phenacyl phosphate was added to the sample and incubated at room temperature for 2 hr. Finally, a chemiluminescent assay of lucigenin was performed. The limit of detection of urinary oxytocin was 2 pg/ml.

### Statistical analysis of demographic, mental health, cognitive, and oxytocin data

Statistical analyses were conducted using the R 4.3.0 software (https://www.r-project.org/). All data were initially assessed for normality and homogeneity of variance, and statistical tests were performed accordingly. Differences in baseline demographics among the groups were analyzed using Student's *t*-test, Welch’s *t*-test, and Chi-square test. The intervention effects on apathy scores, depression scores, MoCA scores, and urinary oxytocin levels were analyzed using a linear mixed-effects model (R packages “lmerTest,” “MuMIn,” and “jtools”). The categorical variables of group (intervention and control) and measurement timing (pre- and post-intervention) were modeled as fixed effects. Individuals were modeled as random intercepts. To adjust for potential temporal/cohort effects over the extended data-collection period, all models included phase (five data-collection periods) as a covariate (Zhang & Volkow, 2023), and urinary oxytocin models additionally adjusted for creatinine. The intervention effect was evaluated based on the interaction term and the results of simple effect analysis. Specifically, an interaction term between group and measurement timing was created and entered into the models. When the interaction was significant, a simple effect analysis was used to examine the extent to which the effect of the measurement timing differed for each group. Effect size (Cohen’s *d*; R packages “emmeans” and “effectsize”) for the group × time interaction and simple effect contrasts were computed from model-based estimated marginal means and standardized by the model residual standard deviation. For the independent variables of interest (group, time, and group × time), *p*-values were corrected using the Benjamini–Hochberg correction to reduce the false discovery rate (FDR) at the .05 level. For the simple effect test (time), corrections for FDR (*p *< .05) were performed using the Benjamini–Hochberg correction.

### Image acquisition

Scanning was performed using a 3T Siemens Magnetom Verio MR scanner (Siemens, Erlangen, Germany). The participant’s head was immobilized using a 12-channel head coil. A gradient echo-planar imaging sequence was used for resting-state functional BOLD images under the following conditions: repetition time, 2,000 ms; echo time, 25 ms; flip angle, 75; number of slices, 39; slice thickness, 3.5 mm; acquisition matrix, 64 × 64; and voxel size, 3.5 × 3.5 × 3.5. The scan lasted approximately 8 min. All participants were instructed to focus continuously on the crosshairs projected onto the screen during scanning. In addition, high-resolution structural images were acquired using an axial T1-weighted magnetization-prepared rapid gradient-echo pulse sequence (field of view, 256 × 256; matrix size, 256 × 256; voxel size, 1 × 1 × 1 mm; 208 slices).

### Image preprocessing and statistical analysis

The CONN toolbox 22v2407 (https://web.conn-toolbox.org/) running in MATLAB R2021a (The MathWorks Inc., United States) was used to preprocess the functional and structural images. The images were preprocessed as follows. The first five scans were removed, and then structural data and resting-state functional data were preprocessed using the default preprocessing pipeline for volume-based analyses. These included functional realignment and unwarping, slice-timing correction, outlier detection, functional direct segmentation and normalization, structural segmentation and normalization, smoothing with an 8-mm Gaussian kernel, and band-pass filtering (0.008–0.09 Hz) (He et al., 2024). For quality control check, one participant in the intervention group and one participant in the control group with excessive head motion ≥3 mm during resting-state fMRI were excluded from the analysis. After preprocessing, the ALFF values of each participant were calculated using the CONN toolbox 22v2407. The power spectrum was obtained using a fast Fourier transform algorithm that converted the time series of each voxel into the frequency domain. Thereafter, a square-root transformation was applied to each frequency in the power spectrum at each voxel. The ALFF value was determined as the root mean square of the BOLD signal after denoising and band-pass filtering between 0.01 and 0.08 Hz (Yang et al., 2007). The ALFF measures across the voxels were then rank sorted and normalized separately for each participant using a Gaussian inverse cumulative distribution function with zero mean and unit variance. Second-level analysis was conducted using the CONN software. A 2 × 2 mixed analysis of variance was used to assess the intervention effects (group × time). Individuals were modeled as random effects. To account for potential confounding factors, covariates included age, sex, education, handedness, and phase, as previously reported (Qi et al., 2019; Zhang & Volkow, 2023). Statistical thresholds were set at *p *< .001, uncorrected for multiple comparisons at the voxel level, and *p *< .05 family-wise error (FWE) corrected for multiple comparisons at the cluster level.

In addition, resting-state FC was assessed using seed-to-voxel analysis. Thereafter, the seeds were set, and regions of interest (ROI) were obtained from the ALFF results. Resting-state FC analysis was performed using the CONN toolbox 22v2407. Functional connectivity strength was represented by Fisher-transformed bivariate correlation coefficients from a weighted general linear model, estimated separately for each seed area and target voxel, modeling the association between their BOLD signal time series. Second-level analysis was conducted using the same method as that used to assess ALFF.

## Results

### Participant flow and baseline demographics

The study flowchart is shown in Figure 1. Of 68 participants, seven withdrew before randomization due to decreased motivation. Eight participants were excluded at the pre-intervention assessment (one with neurological disorders; two with severe sensory/motor impairment; and five with MRI contraindications). Fifty-three participants were then randomized (26 intervention group and 27 control group). During the intervention in the intervention group, one participant was excluded due to a fracture, and one participant withdrew. Two participants in the control group withdrew (one due to decreased motivation and one due to health-related issues). At the post-intervention assessment, one participant in the intervention group and two participants in the control group were excluded due to MRI contraindications. The final analytic sample comprised 44 participants (22 intervention group and 22 control group) with analyzable resting-state fMRI after prespecified quality control.

The baseline demographic data are shown in Table 1. There were no significant between-group differences in age, sex ratio, education, MMSE scores, or time spent on exercise, cognitive activity, paid work, and volunteering. Change in outing frequency from pretest to posttest is shown in Supplementary Results and Supplementary Table 2 (see online supplementary material). These results indicate the demographic equivalence between the groups.

**Table 1.** Baseline demographics of intervention and control groups.

Characteristics	Intervention (*n* = 22)	Control (*n* = 22)	*p* value
**Age (years)**	74.00 (4.46)	74.14 (3.97)	.915
**Sex**			.409
** Male**	5 (22.72)	2 (9.09)	
** Female**	17 (77.27)	20 (90.90)	
**Education (years)**	14.59 (2.48)	13.73 (2.51)	.257
**MMSE (score)**	27.64 (1.62)	27.45 (1.79)	.725
**Exercise (days per week)**	2.05 (2.75)	3.07 (2.30)	.189
**Cognitive activity (days per week)**	1.65 (2.72)	1.13 (2.00)	.467
**Paid work (days per week)**	0.91 (1.74)	0.68 (1.59)	.653
**Volunteering (days per week)**	0.21 (0.50)	0.31 (0.52)	.527

*Note*. MMSE = Mini-mental State Examination; *SD* = standard deviation. Data are presented as the mean (*SD*) or *n* (%). The *p* values for age, education, MMSE, exercise, cognitive activity, paid work, and volunteering are from *t*-tests for group differences. The *p* values for sex ratio are from χ^2^ tests for group differences.

### Intervention effect

#### Psychological measures

**Figure 2.**
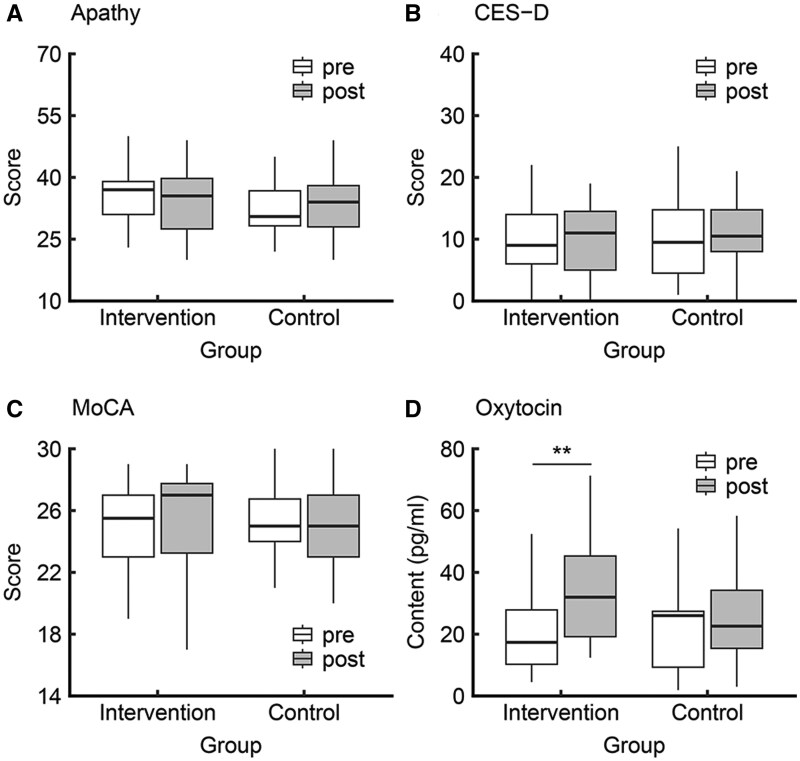
Mental health, MoCA, and oxytocin results from the 3-month dance intervention. (A) Apathy showed a significant group × time interaction; however, within-group pre-post changes were not significant in either group. (B and C) No significant group × time interactions were observed for depressive symptoms or MoCA. (D) Oxytocin showed a significant group × time interaction, with a significant pre-post increase in the intervention group but not in the control group. Box plots show the distribution of individual scores. The horizontal line within each box indicates the median, and the box edges represent the interquartile range. White and grey boxes represent pre- and post-intervention periods, respectively. **p-FDR < .01. FDR = false discovery rate; MoCA = Montreal Cognitive Assessment.

The psychological data are shown in Figure 2A–C. For apathy, the interaction of group and time points was significant (*β* = −0.45, 95% CI [−0.79, −0.10], *R*^2^ = 0.08, *t *= 2.52, *df *= 40.13, *p*-FDR = .048; Cohen’s *d *= −1.11, 95% CI [−2.01, −0.20]). Further investigation with a simple effect analysis indicated no significant time differences in each group (intervention, *β* = −0.22, 95% CI [−0.49, 0.03], *R*^2^ = 0.03, *t *= 1.67, *df *= 19.88, *p*-FDR = .144; Cohen’s *d *= −0.53, 95% CI [−1.16, 0.09]; control, *β*  = 0.21, 95% CI [−0.02, 0.44], *R*^2^ = 0.23, *t *= 1.78, *df *= 19.74, *p*-FDR = .144; Cohen’s *d *= 0.56, 95% CI [−0.06, 1.20]). For the CES-D score, the interaction of group and time points was not significant (*β* = −0.26, 95% CI [−0.71, 0.18], *R*^2^ = 0.02, *t *= 1.17, *df *= 41.43, *p*-FDR = .301; Cohen’s *d *= −0.50, 95% CI [−1.38, 0.36]). For the MoCA scores, the interaction of group and time points was not significant (*β*  =  0.26, 95% CI [−0.23, 0.76], *R*^2^ = 0.03, *t *= 1.04, *df *= 42.25, *p*-FDR = .301; Cohen’s *d *= 0.44, 95% CI [−0.41, 1.31]).

### Oxytocin level

Oxytocin data are shown in Figure 2D. The interaction between group and time point was significant (*β*  =  0.54, 95% CI [0.08, 0.99], *R*^2^ = 0.61, *t *= 2.33, *df *= 40.75, *p*-FDR = .048; Cohen’s *d *= 1.01, 95% CI [0.12, 1.89]). Further investigation with a simple effect analysis indicated a significant time difference in the intervention group (*β* = 0.61, 95% CI [0.29, 0.94], *R*^2^ = 0.57, *t *= 3.70, *df *= 21.13, *p*-FDR = .004; Cohen’s *d *= 1.27, 95% CI [0.62, 1.91]), but not in the control group (*β* = 0.17, 95% CI [−0.15, 0.49], *R*^2^ = 0.68, *t *= 1.02, *df *= 19.94, *p*-FDR = .315; Cohen’s *d *= 0.26, 95% CI [−0.35, 0.88]).

### Resting-state fMRI

**Figure 3.**
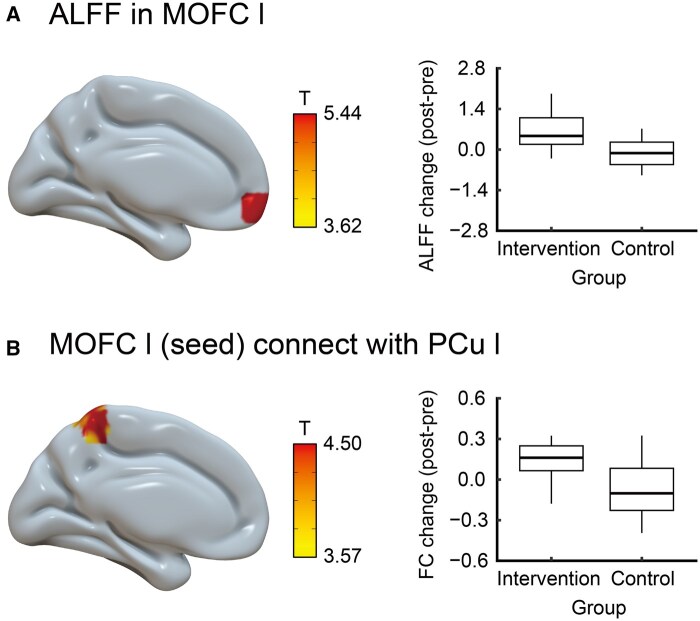
ALFF and resting-state functional connectivity results from the 3-month dance intervention. (A) ALFF of the left medial orbitofrontal cortex is higher post-intervention in the intervention than in the control group. (B) Using ROI in the left medial orbitofrontal cortex as the seed (where the ALFF is greater in the intervention group than in the control group), the post-intervention resting-state FC between the left medial orbitofrontal cortex and left precuneus is stronger in the intervention group than in the control group. ALFF = amplitude of low-frequency fluctuations; FC = functional connectivity; l = left; MOFC = medial orbitofrontal cortex; PCu = precuneus; ROI = region of interest.

The results of the ALFF analysis are shown in Figure 3A and Supplementary Table 3 (see online supplementary material). A significant interaction between group and time point was observed for the ALFF of the left medial orbitofrontal cortex (*F*[1, 37] = 29.07, *p*-FWE = .020). Compared to the control group, the intervention group showed higher ALFF in the left medial orbitofrontal cortex after the intervention. The results of the resting-state FC analysis are shown in Figure 3B and Supplementary Table 4 (see online supplementary material). There was a significant interaction between group and time points in resting-state FC between the left medial orbitofrontal cortex and the left precuneus (*F*[1, 37] = 19.95, *p*-FWE = .006). Compared to the control group, the intervention group showed stronger resting-state FC between the left medial orbitofrontal cortex and left precuneus after the intervention.

## Discussion

The present study investigated the effects of a dance program on mental health, cognitive performance, urinary oxytocin levels, and resting-state neural activity in older adults with SCD. First, depressive symptoms and cognition did not change significantly. Second, changes in apathy over time differed between groups; however, neither group’s pre-post change reached significance. Third, the intervention group showed a greater pre-post increase in urinary oxytocin levels than the control group, with a significant increase from the pretest to posttest in the intervention group. Finally, neuroimaging measures showed a significant between-group difference in pre-post change: ALFF in the left medial orbitofrontal cortex and resting-state FC between the left medial orbitofrontal cortex and left precuneus increased more from pre- to post-intervention in the intervention group than in the control group. These findings suggest that for individuals with SCD, a 12-week dance training program may improve spontaneous neural activity and resting-state FC in default mode regions, along with an increase in oxytocin levels.

At the behavioral level, apathy exhibited a significant between-group difference in change over time, with no statistically significant change in either group. In contrast, ­Sanprakhon et al. (2025) reported that among older adults with MBI, a 7-week multicomponent program combining traditional Thai folk dance with cognitive stimulation therapy was associated with a reduction in behavioral impairment, including apathy. This discrepancy might be caused by differences in the intervention content; our program was a 12-week dance-only intervention. In addition, the modest sample size likely limited the precision and power to detect small effect sizes.

Despite the lack of a clear intervention effect on apathy, the intervention group showed increased urinary oxytocin levels after the intervention. Oxytocin is involved in prosocial behaviors, such as interpersonal trust, relationships, and approach/avoidance (Harari-Dahan & Bernstein, 2014; Harvey, 2020). Oxytocin is produced in the hypothalamus and projects to the prefrontal cortex and limbic system via the pituitary gland (Harvey, 2020; Quintana & Alvares, 2016). Among the various brain regions that receive oxytocinergic inputs, oxytocin changes in the prefrontal cortex appear to be particularly relevant for the facilitation of social bonding by enhancing cognitive control in the regulation of emotions (Bos et al., 2012). Furthermore, oxytocin levels were found to be positively correlated with activity in the prefrontal cortex and amygdala during a social judgment task (Gordon et al., 2013), suggesting a crucial role for the prefrontal-amygdala oxytocinergic circuit in socioemotional processing. Combined with previous findings that dance enhances emotional cohesion and inclination for social activities through affective transmission (Hagen & ­Bryant, 2003; Nadasen, 2008), such effects could be related to enhanced oxytocinergic function.

Furthermore, consistent with the elevated oxytocin level, the intervention group showed increased ALFF in the left medial orbitofrontal cortex and resting-state FC between the left medial orbitofrontal ROI and the left precuneus after the intervention, suggesting strengthened medial orbitofrontal-precuneus integration. These findings suggest that dance training may induce neural functional benefits in two key ways. First, the coupling between the anterior (i.e., medial prefrontal cortex) and posterior (i.e., precuneus) regions within the default mode network declines with age and relates to cognitive decline in healthy older adults (Andrews-Hanna et al., 2007; Ng et al., 2016). This motivates the hypothesis that sustained engagement in dance could mitigate such age-related decoupling. Second, a previous study indicated reduced medial orbitofrontal-precuneus FC in older adults with SCD compared to those without SCD (Li et al., 2024). Thus, the SCD-related reduction in medial orbitofrontal-precuneus FC might be viewed as an extension of the age-related anterior-posterior default mode network decoupling observed in healthy older adults. The present findings situate our effects within a continuum from healthy aging to SCD. They also suggest that dance training may partially improve or compensate for this decoupling in individuals with SCD.

The medial orbitofrontal cortex and precuneus also play important roles in social behavior, emotional processing, and self-awareness (Gangopadhyay et al., 2021; Gilboa et al., 2004; Porcelli et al., 2019; Ye et al., 2019). Golde et al. (2019) reported that reduced activation of the ventromedial prefrontal cortex, including the medial orbitofrontal cortex, is associated with social loneliness. This suggests that a functional reduction in this region may lead to diminished emotional and social engagement. Moreover, a previous study reported that lower FC in the medial prefrontal cortex, including the medial orbitofrontal cortex and precuneus, was associated with negative automatic thoughts (Yeo et al., 2024), suggesting that such an association may contribute to low self-esteem (Rolls et al., 2020). The previous and present findings support that dance training may enhance social-affective and self-referential processing by strengthening medial orbitofrontal activity and FC with the precuneus in individuals with SCD. This neural enhancement within the default-mode network may eventually contribute to a more positive self-perception and socioemotional well-being in the long term.

Although the intervention affected oxytocin levels and brain activity, it did not improve depression or cognition. The failure to detect a significant effect on depression may be due to uncontrolled lifestyle factors, such as the frequency of exercise and cognitive activities before and during the intervention (Table 1 and Supplementary Table 1 [see online supplementary material]). Our participants had a generally high level of daily activity and motivation for well-being. These characteristics may have weakened the effects of dance training on depression. In addition, the failure to detect a significant effect on cognition may be explained by a ceiling effect owing to the high pretest scores of our participants. In the study by Qi et al. (2019), the mean MoCA score was significantly improved from 22.6 at pretest to 24.3 at posttest in the dance intervention group with MCI. In contrast, in our intervention group, the mean MoCA score was numerically higher at posttest (25.5) than at pretest (24.7), but this change was not statistically significant. Older adults with lower cognitive performance pre-intervention tend to benefit most from dance training (Kattenstroth et al., 2013). This may explain the limited improvement observed in our intervention group with high pretest MoCA scores. Another possible reason is the difference in the intervention frequency. In our study, dance lessons were only given once a week, whereas they were given three times weekly in the study by Qi et al. (2019). These differences in intervention frequency may have contributed to the failure to observe significant improvements in the MoCA scores in our study.

Our study had limitations. First, our study had a small sample size owing to recruitment challenges during the coronavirus disease 2019 pandemic, and this may have limited the statistical power. Second, some participants had an active lifestyle (i.e., engaging in physical and cognitive activities) before and during the intervention (Supplementary Table 3 [see online supplementary material]). Therefore, the findings may not be generalizable to older adults with SCD who have a less active lifestyle, potentially limiting the applicability of the intervention to other populations. Third, the sample included only a few male participants (7/44 overall [15.9%]: 5/22 in the intervention group [22.7%] and 2/22 in the control group [9.1%]), which may limit the generalizability across sexes. Fourth, the dance sessions were held once a week by design to reflect a frequency that older adults could practicably integrate into daily life. This pragmatic schedule was designed to enhance feasibility in community settings, whereas the modest dose may have attenuated measurable effects. Finally, we did not measure prosocial behavior (i.e., trust-related behavioral tasks and empathy-related questionnaires). Future research should investigate whether the observed oxytocin and resting-state neural activity changes translate into interpersonal behaviors.

## Conclusion

To the best of our knowledge, this study is the first to provide evidence of neurobiological improvements through dance training in individuals with SCD. Dance intervention increases urinary oxytocin levels, spontaneous neural activity in the left medial orbitofrontal cortex, and resting-state FC with the left precuneus in older adults with SCD, suggesting enhanced intrinsic brain activity and network integration related to self-referential and socioemotional processes. At the behavioral level, depressive symptoms and cognitive function showed no clear intervention-related changes, and apathy exhibited a different temporal pattern without a clear intervention effect. This pattern suggests that neurobiological effects may precede detectable changes in mental health and cognition. Detecting such changes may require a greater training dose or longer duration, or participants with lower baseline activity levels. Taken together, dance training is related to functional advantages in the neural system involving the medial orbitofrontal cortex, along with potential oxytocinergic benefits. These findings provide new insights into the mechanisms through which dance training contributes to socioemotional resilience and brain health in older adults with SCD.

## Supplementary Material

igaf129_Supplementary_Data

## Data Availability

The data will be made available upon reasonable request, as the authors have not yet completed their planned analyses of the data set. This study was preregistered with the University Hospital Medical Information Network (UMIN) Clinical Trials Registry [ID: UMIN000047106; https://center6.umin.ac.jp/cgi-open-bin/ctr/ctr_view.cgi?recptno=R000053736].
