# Supplementary material for: Effects of dance training on oxytocin secretion and neural activity in older adults with subjective cognitive decline
Source: Innov Aging. 2025 Nov 14;10(1):igaf129. doi: 10.1093/geroni/igaf129 (PMC12759060; doi:10.1093/geroni/igaf129)
Supplement: igaf129_Supplementary_Data [file igaf129_supplementary_data.zip › innage suppl Yamashita, Toyoshima, et al.docx]

***Innovation in Aging* Supplementary Material: Yamashita et al. Effects of dance training on oxytocin secretion and neural activity in older adults with subjective cognitive decline.**

Supplementary Methods. Outing frequency questionnaire and statistical analysis

Supplementary Results. Outing frequency

Supplementary Table 1. Participant counts by data-collection phase

Supplementary Table 2. Outing days per week by group at pretest and posttest

Supplementary Table 3. ALFF analysis results

Supplementary Table 4. Seed-to-voxel analysis results

**Supplementary Methods. Outing frequency questionnaire and statistical analysis**

The outing frequency questionnaire was administered using an actual month-long calendar for the month preceding the assessment. An outing day was defined as ≥2 hours out of home for leisure activities, volunteering, paid work, shopping, or visiting relatives. Each calendar day was coded “1” if any outing occurred and “0” otherwise; multiple outings on the same day were counted as one. The outcome was outing days per week.

Statistical analyses were conducted using the R 4.3.0 software. Differences in outing days per week across group (intervention and control) and time (pre and post) were analyzed using a two-way mixed ANOVA (R packages “afex” and “effectsize”). Group and time were modeled as fixed effects, and repeated measures over time were accommodated by treating individuals as the random effect. The primary test was the group × time interaction. Statistical significance was set at *α* = 0.05.

**Supplementary Results. Outing frequency**

The outing days per week are shown in Supplementary Table 3. The interaction of group and time points was not significant (*F*[1, 42] = 0.19, *p* = 0.669, partial *η*^2^ = 0.004). The main effect of group was not significant (*F*[1, 42] = 1.51, *p* = 0.226, partial *η*^2^ = 0.035). The main effect of time was also not significant (*F*[1, 42] = 2.41, *p* = 0.128, partial *η*^2^ = 0.054). These results indicate stable community mobility over time and no differential change between groups.

**Supplementary Table 1.** Participant counts by data-collection phase

| Five data correction periods | Randomized *n*  (intervention/control) | Analyzed *n*  (intervention/control) |
| --- | --- | --- |
| Phase 1 (May to September 2022) | 3 (1 / 2) | 1 (0 / 1) |
| Phase 2 (July to December 2022) | 13 (6 / 7) | 9 (4 / 5) |
| Phase 3 (October 2022 to February 2023) | 11 (5 / 6) | 10 (5 / 5) |
| Phase 4 (February to July 2023) | 17 (11 / 6) | 16 (10 / 6) |
| Phase 5 (October 2023 to February 2024) | 9 (3 / 6) | 8 (3 / 5) |

Notes. Counts reflect randomized participants; pre-randomization withdrawals are not attributed to either arm (*n* = 7).

**Supplementary Table 2.** Outing days per week by group at pretest and posttest

|  | Intervention  (*n* = 22) | Control  (*n* = 22) |
| --- | --- | --- |
| Pretest | 3.57 (1.34) | 4.25 (2.02) |
| Posttest | 3.93 (1.50) | 4.45 (1.98) |

Notes. SD = standard deviation. Data are presented as the mean (*SD*).

**Supplementary Table 3.** ALFF analysis results

| Region | MNI coordinates (X, Y, Z) | Cluster size | t value | *p*-FWE |
| --- | --- | --- | --- | --- |
| MOFC l | -8, 56, -10 | 90 | 5.39 | 0.020 |

Notes. ALFF = amplitude of low-frequency fluctuations**;** FEW = family wise error; l, left; MNI = Montreal Neurological Institute; MOF = medial orbitofrontal cortex.

**Supplementary Table 4.** Seed-to-voxel analysis results

| Region | MNI coordinates (X, Y, Z) | Cluster size | t value | *p*-FWE |
| --- | --- | --- | --- | --- |
| PCu l | -16, -46, 66 | 217 | 4.47 | 0.006 |

Notes. FEW = family wise error; l, left; MNI = Montreal Neurological Institute; PCu = precuneus.
